# Workshop Report on Global Harmonization of Enterovirus Vaccines

**DOI:** 10.3201/eid2604.191273

**Published:** 2020-04

**Authors:** Tzu-Yu Weng, Hua Yen, Kutub Mahmood, Javier Martin, Min-Shi Lee

**Affiliations:** National Health Research Institutes, Zhunan, Taiwan (T.-Y. Weng, H. Yen, M.-S. Lee);; Center for Vaccine Innovation and Access, PATH, Seattle, Washington, USA (K. Mahmood);; National Institute for Biological Standard and Control, Potters Bar, UK (J. Martin)

**Keywords:** conference, enterovirus vaccines, vaccines, viruses, Sabin strain-derived IPV, sIPV, enterovirus A71, EV-A71, poliovirus, oral poliovirus vaccine, OPV, inactivated poliovirus vaccine

The Asia-Pacific Network for Enterovirus Surveillance in the National Health Research Institutes hosted a workshop on May 2, 2019, to discuss the harmonization of 2 licensed enterovirus vaccines, poliovirus and enterovirus A71 (EV-A71), especially regarding the standardization of vaccine antigens (Appendix). Speakers from the UK National Institute for Biologic Standard and Control (NIBSC), US PATH, industries, and regulatory agencies shared their experiences.

Sabin strain–derived live-attenuated oral poliovirus vaccines (OPV) and wild strain–derived inactivated poliovirus vaccines (IPV) have been widely used. Since wild-type 2 poliovirus was eradicated, bivalent OPV (only type 1 and 3) replaced trivalent OPV. Wild-type 3 is almost eradicated, and major efforts are under way to eradicate type 1. In addition, wild strains are not feasible to produce conventional IPV (cIPV) after wild polioviruses are eradicated. Therefore, the World Health Organization (WHO) recommends the development of Sabin strain–derived IPV (sIPV) for the endgame plan of global poliovirus eradication programs. Because WHO international standard (IS) antigen for quantifying cIPV antigens could not be effectively used to quantify sIPV, new reagents based on sIPV need to be established.

Three EV-A71 vaccines have been licensed in China, and 2 EV-A71 vaccine candidates were evaluated in phase III trials in Taiwan. Experience with IPV indicates that establishing WHO IS antigens is critical for global harmonization of EV-A71 vaccines. Thus, NIBSC and the China National Institute of Food and Drug Control (NIFDC) coordinated an international study for the first IS antigen of EV-A71 vaccines. Some participants in the international study shared their results in the workshop. This report summarizes outcomes of presentations and discussion.

## Global Development of sIPV

In 2018, global demand for IPV was ≈90 million doses, but only ≈70 million doses of IPV were delivered, suggesting a potential shortage of IPV. To mitigate the IPV shortage for vaccination campaigns (catch-up or outbreak responses), WHO recommended that a fractional dose (one fifth of full IPV dose) could be administered intradermally (https://www.who.int/immunization/sage/meetings/2018/april). Regarding the global development of sIPV, 4 organizations have obtained marketing approval for sIPV, but D-antigen units of these 4 sIPV differ. Therefore, PATH is collaborating with WHO and NIBSC to develop standard antigens and antibody reagents to quantify D-antigen units of sIPV. According to the current WHO plan, IPV vaccination will continue for at least 10 years after poliovirus eradication. High-income countries will use combination vaccines containing IPV for a longer period.

## Global Harmonization of sIPV

Potency of cIPV is evaluated using 2 methods: 1) in vitro ELISA to measure D-antigen unit and 2) in vivo rat immunogenicity to measure postvaccination serum neutralization antibody titers. For in vitro ELISA, an IS antigen for cIPV (IS 12/104) has been established but is not suitable for measuring D-antigen unit of sIPV. Consequently, NIBSC initiated the international collaboration study to establish the first IS of sIPV (*1*). It is essential to understand the correlation between in vitro and in vivo vaccine potencies for different sIPV products and ideally link both to results in human studies.

## Global Harmonization of EV-A71 Vaccines

Three EV-A71 vaccines (genotype C4) were licensed in China, and these 3 vaccines have different dose units. Therefore, NIFDC, China established a national standard to harmonize the measurement of EV-A71 vaccine antigens (*2*). In Taiwan, 2 EV-A71 vaccine candidates (genotype B4) were evaluated in phase III trials, and doses of these 2 candidates were determined by quantifying total proteins. In 2015, NIBSC and NIFDC established the first IS for human EV-A71 antiserum for measuring serum neutralizing antibody titers (*3*). Likewise, on the basis of prior experiences with IPV, establishing potency assays of EV-A71 vaccines is pivotal for global harmonization, including in vitro ELISA for measuring D-antigen units and in vivo animal immunogenicity for measuring postvaccination serum neutralization antibody titers.

## In Vitro ELISA for Measuring D-Antigen Units of Inactivated EV-A71 Vaccines

Four potential IS candidates (2 genotype C4 products from China and 2 genotype B4 products from Taiwan) were evaluated in an international collaboration study. NIBSC developed lyophilization protocols to optimize the stability of IS candidates and established an ELISA using mouse monoclonal antibody (CT11F9) as detection antibody and rabbit polyclonal antibody as capture antibody. Fourteen laboratories joined the international study, and most of them established in-house ELISA using different mouse monoclonal antibodies and rabbit polyclonal antibodies. EV-A71 vaccines contain both empty and full virus particles with different structural conformation (*4*–*6*), which is not the case for IPV products. Therefore, it may be necessary to understand the relative contribution of these 2 viral forms to the antigenic and immunogenic properties of different EV-A71 vaccines. Moreover, establishing the correlation between in vitro ELISA and in vivo animal immunogenicity for both full and empty EV-A71 virus particles is critical. The 2 different particles need to be obtained by purification and heat-treatment, which enables transition from full to empty virus particles. In addition, clarifying the necessity of establishing IS for different EV-A71 genotypes is important.

## In Vivo Animal Immunogenicity

Mice, rats, and rabbits have been used to evaluate immunogenicity of inactivated EV-A71 vaccines, but standard protocols for these animal models have not been established. Previous studies have detected antigenic variations among EV-A71 genogroups A, B, and C and correlated cross-reactive antibody responses with EV-A71 infections in rabbits and children (*7*,*8*). Correlating cross-reactive antibody responses with EV-A71 vaccination in animals and children is desirable. Previous IPV studies had shown that rats were used to evaluate immunogenicity with multiple dose units (1, 0.5, 0.25, 0.125, and 0.0625 human dose units) (*9*,*10*). Therefore, similar animal studies should be conducted for inactivated EV-A71 vaccines.

Appendix Agenda for a workshop on global harmonization of enterovirus vaccines.
