# Supplementary material for: Workshop Report on Global Harmonization of Enterovirus Vaccines
Source: Emerg Infect Dis. 2020 Apr;26(4):e191273. doi: 10.3201/eid2604.191273 (PMC7101126; doi:10.3201/eid2604.191273)
Supplement: Appendix — Agenda for a workshop on global harmonization of enterovirus vaccines. [file 19-1273-Techapp-s1.pdf]

Article DOI: https://doi.org/10.3201/eid2604.191273

Workshop Report on Global Harmonization of Enterovirus Vaccines

Appendix

Workshop Agenda

May 2[ed], 2019

Venue: Howard Civil Service International House, Room 101			
**AGENDA 議程**			
**I. Global Harmonization of Polio Vaccines**			
**TIME**	**TOPIC**	**SPEAKER**	**MODERATOR**
**08:30-09:00**	Registration		
**09:00-09:10**	Opening Remark	Dr. Peter Wu-Shou Chang (張武修委員) Member of the Control Yuan, Taiwan<br>Dr. Kung-Yee Liang (梁賡義院長) President, NHRI, Taiwan	
**09:10-09:30**	Polio Vaccines and Poliovirus eradication	Dr. Kutub Mahmood PATH, USA	Dr. Mei-Shang Ho (何美鄉教授) Academia Sinica, Taiwan
**09:30-10:00**	Global harmonization of Sabin IPV with established International standard	Dr. Kutub Mahmood PATH, USA<br>Dr. Javier Martin NIBSC, UK	
**10:00-10:15**	*Coffee Break*		
**II. Global Harmonization of EV-A71 Vaccines**			
**10:15-10:35**	Global harmonization of EV-A71 vaccines: overview	Dr. Javier Martin NIBSC, UK	Dr. Mei-Shang Ho (何美鄉教授) Academia Sinica, Taiwan
**10:35-11:05**	EV-A71 vaccine animal model: experience sharing	Mr. Charles Jia-Chuan Hsu (許家銓) FDA, Taiwan	
**11:05-11:20**	Quantification of EV-A71 vaccine antigens: experience sharing	Dr. Tzu-Yu Weng (翁子玉) NHRI, Taiwan	Prof. Oliver Yoa-Pu Hu, (胡幼圃講座教授) Taipei Medical University, Taiwan
**11:20-11:40**	Quantification of EV-A71 vaccine antigens: perspectives from Adimmune	Dr. Charlie Chen (陳昌立) Adimmune, Taiwan	
**11:40-12:00**	Quantification of EV-A71 vaccine antigens: experience sharing	Dr. Scott Lee (李祥吉) Medigen, Taiwan	
**12:00-12:30**	Global harmonization of EV-A71	Dr. Javier Martin	

	vaccines: future plan	NIBSC, UK	
**12:30-14:00**	*Group Photo & Lunch Hour*		
**III. Development of Multivalent Enterovirus Vaccines**			
**14:00-14:30**	Enterovirus surveillance: new technology for old problems	Dr. Javier Martin<br>NIBSC, UK	Dr. Li-Min Huang<br>(黃立民主任)<br>NTU, Taiwan
**14:30-15:00**	Asia-Pacific Network for Enterovirus Surveillance (APNES)	Dr. Fang-Lin Kuo (郭方琳)<br>NHRI, Taiwan	
**15:00-15:30**	Development of multivalent enterovirus vaccines: selection of serotypes and vaccine strains	Dr. Min-Shi Lee (李敏西)<br>NHRI, Taiwan	
**15:30-16:00**	*Coffee Break*		
**16:00-17:00**	Panel Discussion		Dr. Tzou-Yien Lin<br>(林奏延董事長)<br>Chair person, Board of Directors, NHRI, Taiwan
**Group Photo**			
